# Zika Virus Promotes Neuronal Cell Death in a Non-Cell Autonomous Manner by Triggering the Release of Neurotoxic Factors

**DOI:** 10.3389/fimmu.2017.01016

**Published:** 2017-08-23

**Authors:** Isabella G. Olmo, Toniana G. Carvalho, Vivian V. Costa, Juliana Alves-Silva, Carolina Z. Ferrari, Tatiane C. Izidoro-Toledo, Juliana F. da Silva, Antonio L. Teixeira, Danielle G. Souza, Joao T. Marques, Mauro M. Teixeira, Luciene B. Vieira, Fabiola M. Ribeiro

**Affiliations:** ^1^Department of Biochemistry and Immunology, Institute of Biological Sciences (ICB), Universidade Federal de Minas Gerais (UFMG), Belo Horizonte, Brazil; ^2^Department of Pharmacology, ICB, UFMG, Belo Horizonte, Brazil; ^3^Institute of Education and Research Santa Casa, Belo Horizonte, Brazil; ^4^Neuropsychiatry Program, Department of Psychiatry and Behavioral Science, UT Health, Houston, TX, United States; ^5^Department of Microbiology, ICB, UFMG, Belo Horizonte, Brazil

**Keywords:** Zika virus, *N*-methyl-d-aspartate receptors, GluN2B, tumor necrosis factor-α, interleukin-1β

## Abstract

Zika virus (ZIKV) has recently caused a worldwide outbreak of infections associated with severe neurological complications, including microcephaly in infants born from infected mothers. ZIKV exhibits high neurotropism and promotes neuroinflammation and neuronal cell death. We have recently demonstrated that *N*-methyl-d-aspartate receptor (NMDAR) blockade by memantine prevents ZIKV-induced neuronal cell death. Here, we show that ZIKV induces apoptosis in a non-cell autonomous manner, triggering cell death of uninfected neurons by releasing cytotoxic factors. Neuronal cultures infected with ZIKV exhibit increased levels of tumor necrosis factor-α (TNF-α), interleukin-1β (IL-1β), and glutamate. Moreover, infected neurons exhibit increased expression of GluN2B and augmented intracellular Ca^2+^ concentration. Blockade of GluN2B-containing NMDAR by ifenprodil normalizes Ca^2+^ levels and rescues neuronal cell death. Notably, TNF-α and IL-1β blockade decreases ZIKV-induced Ca^2+^ flux through GluN2B-containing NMDARs and reduces neuronal cell death, indicating that these cytokines might contribute to NMDAR sensitization and neurotoxicity. In addition, ZIKV-infected cultures treated with ifenprodil exhibits increased activation of the neuroprotective pathway including extracellular signal-regulated kinase and cAMP response element-binding protein, which may underlie ifenprodil-mediated neuroprotection. Together, our data shed some light on the neurotoxic mechanisms triggered by ZIKV and begin to elucidate how GluN2B-containing NMDAR blockade can prevent neurotoxicity.

## Introduction

Zika virus (ZIKV) is an arthropod-borne virus (arbovirus) from the *Flavivirus* genus within the *Flaviviridae* family that was first isolated in 1947 from a rhesus monkey in the Ziika forest in Uganda (1). Only 14 sporadic and benign cases of ZIKV infection were documented in humans prior to the first large epidemic outbreak, which took place on the Island of Yap in 2007 (2, 3). This was followed by a major ZIKV outbreak in the French Polynesia from October 2013 to April 2014 (4). Since 2015, 76 countries and territories around the world have reported mosquito-borne ZIKV transmission, triggering an ongoing epidemic in South America, where Brazil was the main affected country (5). These recent ZIKV outbreaks have been associated with severe neurological complications, including microcephaly and congenital neurological malformations in infants born from infected mothers (6–9), as well as Guillain–Barré syndrome in adults (10–12). As a consequence, in February 2016, the World Health Organization declared that the ZIKV outbreak was a public health emergency of international concern (13). ZIKV was detected in the placenta and amniotic fluid of two pregnant women whose fetuses had been diagnosed with microcephaly (14–16), indicating that ZIKV can cross the placental barrier. The virus has also been found in the brains and retinas of microcephalic fetuses (16–18). ZIKV exhibits high neurotropism and can promote neuroinflammation and neurodegeneration, which is the main correlate of ZIKV-associated neurological changes (17, 19–21).

We have recently demonstrated that *N*-methyl-d-aspartate receptor (NMDAR) blockade by memantine could prevent ZIKV-induced cell death of primary cultured corticostriatal neurons (22). Type I interferon receptor-deficient mice (IFNα/βR^−/−^) infected with ZIKV exhibited high levels of neurodegeneration, microgliosis, and inflammatory response (22). Importantly, treatment of ZIKV-infected IFNα/βR^−/−^ mice with 30 mg/kg memantine was efficient to prevent microglia proliferation and neurodegeneration in all brain substrates tested, including prefrontal and motor cortex, striatum, and hippocampus (22). Moreover, memantine treatment was effective to prevent ZIKV-induced increase in total and differential blood leukocyte counts (22). Despite these very promising results, little is known on how ZIKV induces neurodegeneration and on how NMDAR blockade rescues ZIKV-induced neuronal cell loss.

In the present work, we examined whether the release of neurotoxins by ZIKV-infected cells could contribute to death of uninfected nearby neurons, triggering apoptosis in a non-cell autonomous manner. Neuronal cultures infected with ZIKV exhibited increased levels of tumor necrosis factor-α (TNF-α), interleukin-1β (IL-1β), and glutamate. The increase in TNF-α and IL-1β production facilitated NMDAR sensitization, thereby increasing Ca^2+^ entry into the cell and promoting excitotoxicity. Blockade of GluN2B-containing NMDARs by ifenprodil decreased intracellular Ca^2+^ concentration and rescued neuronal cell death. In addition, ZIKV-infected cultures treated with ifenprodil exhibited augmented activation of extracellular signal-regulated kinase (ERK) and cAMP response element-binding protein (CREB), which may contribute to ifenprodil-mediated neuroprotection. Together, these data shed some light on the neurotoxic mechanisms triggered by ZIKV and begin to elucidate how GluN2B-containing NMDAR blockade can prevent neurotoxicity.

## Materials and Methods

### Materials

Neurobasal medium, N2 and B27 supplements, GlutaMAX, penicillin and streptomycin, Live/Dead viability assay, TRIzol™, Power SYBR™ Green PCR Master Mix, anti-rabbit Alexa Fluor 488 antibody, anti-mouse Alexa Fluor 546, and DAPI (4′,6-Diamidino-2-Phenylindole, Dihydrochloride) were purchased from Thermo Fisher Scientific. (1R*, 2S*)-*erythro*-2-(4-Benzylpiperidino)-1-(4-hydroxyphenyl)-1-propanol hemi-(DL)-tartrate (Ifenprodil) were purchased from Tocris Cookson Inc. Horseradish peroxidase conjugated anti-rabbit IgG secondary antibody was from BioRad. Western Blotting ECL Prime detection reagents were from GE Healthcare and Immobilon Western Chemiluminescent HRP Substrate was from Millipore. Anti-phospho-ERK1/2 (Thr202/Tyr204), anti-ERK1/2, and anti-phospho-CREB (Ser133) rabbit antibodies and anti-CREB mouse antibody were purchased from Cell Signaling. All other biochemical reagents were purchased from Sigma-Aldrich.

### Animals

C57BL/6 mice (25–30 g) were purchased from the animal facility (CEBIO) from the Universidade Federal de Minas Gerais (UFMG). Mice were housed in an animal care facility at 23°C on a 12-h light/12-h dark cycle with food and water provided *ad libitum*. This study was carried out in accordance with the recommendations of the Brazilian Government (law 11794/2008a) and approved by the Committee on Animal Ethics of the UFMG (CEUA/UFMG, permit protocol no. 242/2016).

### Virus

A low-passage-number clinical isolate of ZIKV (HS-2015-BA-01), isolated from a viremic patient with symptomatic infection in Bahia State, Brazil, in 2015, was used. The complete genome of the virus is available at GenBank under the accession no. KX520666. Virus stocks were propagated in C6/36 *Aedes albopictus* cells and titrated as described previously (23).

### Neuronal Primary Cultures Preparation

Neuronal cultures were prepared from the cerebral cortex and striatal regions of C57BL/6 wild-type mouse embryo brains, embryonic day 15 (E15). After dissection, the brain tissue was submitted to trypsin digestion followed by cell dissociation using a fire-polished Pasteur pipette. Neuronal cells were plated onto poly-l-ornithine-coated dishes in Neurobasal^®^ medium supplemented with N2 and B27^®^ supplements, 2 mM GlutaMAX™, and penicillin and streptomycin (50 µg/mL each), and cultured *in vitro* for 5 days at 37°C and 5% CO_2_ in a humidified incubator.

### Viral Infection and Neuronal Treatment

Primary neuronal cultures were incubated with either ZIKV (MOI of 0.1) or C6/36 supernatant (MOCK) for 1 h (adsorption period). After that, residual virus was removed and replaced by supplemented neurobasal medium. Kinetic experiments evaluating the effects of ZIKV on primary neurons were performed after 12, 24, 36, 48, and 72 h of ZIKV infection. When MOCK- or ZIKV-infected neuronal cultures were treated with ifenprodil, etanercept, or IL1-RA, drugs were added immediately after viral infection and replenished every 24 h in experiments lasting between 36 and 72 h. Following this incubation, neuronal cultures were assessed for cell death/survival, glutamate release, and [Ca^2+^]_i_ quantitation, or processed for immunofluorescence, quantitative RT-PCR (RT-qPCR), and western blot analyses.

### Cell Death Assay

Neuronal cells death was determined by *LIVE/DEAD Cell Viability Assays*, as previously described (24), at different time points after infection (as indicated in each *figure legend*). Briefly, MOCK- or ZIKV-infected neurons, submitted to different drug treatments, were stained with 2 µM calcein acetoxymethyl ester (AM) and 2 µM ethidium homodimer-1 for 15 min and the fractions of live (calcein AM positive) and dead (ethidium homodimer-1 positive) cells were determined. Neurons were visualized and imaged in a fluorescence microscope, *FLoid*^®^ *Cell Imaging Station* (Thermo Scientific). A minimum of 150 cells were analyzed per well in triplicate using ImageJ software. Dead cells were expressed as a percentage of the total number of cells.

### Glutamate Release Assay

Glutamate release by primary cultured neurons was measured indirectly by the fluorescence increase due to the production of NADPH in the presence of glutamate dehydrogenase type II and NADP^+^ (25). Neuronal cultures, challenged with ZIKV or MOCK for 48 h, were incubated with 1 mM CaCl_2_ and 1 mM NADP^+^ in Hank’s balanced salt solution (HBSS) and analyzed in a spectrofluorometer (Synergy 2, BioTek^®^ Instruments, Inc.) using excitation wavelength of 360 nm and emission of 450 nm. Glutamate dehydrogenase (50 units per well) was added to each well after 5 min, and readings were restarted until the fluorescence reached balance (approximately 5 min). Calibration curves were carried out in parallel with the addition of known amounts of glutamate (5 nM/µL) to the reaction medium. Glutamate levels were normalized to the total amount of protein per well. Experimental data are expressed as percentage, taking basal glutamate release (time 0) as 100%. The experiments were performed at 37°C in triplicate well for each condition.

### Measurement of Intracellular Ca^2+^ Concentration

Neuronal cultures, MOCK- or ZIKV-infected and submitted to different drug treatments, as described in the *Figure Legend*, were loaded with 0.2 µM Fura-2 AM for 20 min at 37°C. Neurons were washed with HBSS and illuminated with alternating 340- and 380-nm light, with the 510 nm emission detected using a PTI spectrofluorimeter (Synergy 2, BioTek^®^ Instruments, Inc.). At the end of each experiment, sodium 10% dodecyl sulfate (SDS) (0.1% final) was added to obtain *R*_max_ followed by 3 M Tris + 400 mM EGTA (pH 8.6) for *R*_min_. All experiments were performed in triplicate wells for each condition.

### Immunoblotting

12 or 24 h following MOCK or ZIKV infection, neurons, treated or not with 0.01 µM ifenprodil, were lysed in RIPA buffer (0.15 M NaCl, 0.05 M Tris–HCl, pH 7.2, 0.05 M EDTA, 1% non-idet P40, 1% TritonX-100, 0.5% sodium deoxycholate, 0.1% SDS) containing SIGMAFAST™ Protease Inhibitor Cocktail Tablets. 100 µg of total cellular protein for each sample was subjected to SDS-PAGE, followed by electroblotting onto nitrocellulose membranes. Membranes were blocked with 5% BSA in wash buffer (150 mM NaCl, 10 mM Tris–HCl, pH 7.4, and 0.05% Tween 20) for 1 h and then incubated with either rabbit anti-phospho CREB (1:500) or rabbit anti-phospho ERK (Thr202/Thr204) (1:1,000) antibodies in wash buffer containing 3% BSA overnight at 4°C. Membranes were rinsed three times with wash buffer and then incubated with secondary peroxidase conjugated anti-rabbit IgG antibody diluted 1:5,000 in wash buffer containing 3% BSA for 1 h. Membranes were rinsed three times with wash buffer, incubated with ECL prime western blotting detection reagents, and scanned and analyzed by ImageQuant LAS 4000 (GE Healthcare). Antibodies were then stripped and membranes were incubated with either mouse anti-CREB (1:700) or rabbit anti-ERK1/2 (1:1,000) antibodies overnight at 4°C and probed with secondary anti-mouse IgG antibody diluted 1:2,500 or anti-rabbit IgG antibody 1:5,000 to determine total CREB and ERK1/2 expression, respectively. Non-saturated, immunoreactive CREB and ERK1/2 bands were quantified by scanning densitometry. Immuno-band intensity was calculated using ImageJ software, and the number of pixels of CREB and ERK1/2 phospho bands was divided by the number of pixels of total CREB and ERK1/2, respectively, to normalize phosphorylation levels of kinases to total kinase expression.

### Measurement of Cytokine Concentrations (ELISA)

Cytokine concentration (IL-1β and TNF-α) was measured in the supernatant of neuronal cultures at 12, 24, or 48 h following MOCK or ZIKV infection. Cytokine measurement was performed using commercially available antibodies and according to the procedures supplied by the manufacturer (R&D Systems, Minneapolis, MN, USA). Briefly, 96-well plates were sensitized with capture antibody and incubated overnight. Plates were then washed and blocked with 200 µL of 1% BSA solution for 60 min. After a second wash, 100 µL of the standard curve (serially diluted) and each sample were added to wells in duplicate and a new incubation was performed on the plate shaker at agitation of 300 rpm, 37°C, for 2 h. Following a further wash, 100 µL of the detection antibody was added and plates were re-incubated for 2 h. Between each wash, streptavidin was added for 20 min, followed by substrate o-phenylenediamine dihydrochloride (OPD Sigma) addition for 30 min. Reaction was stopped by the addition of 100 µL of the stop solution (H_2_SO_4_). The standard curve and samples were read in a spectrophotometer at 490 nm. The detection limit of each kit is 4–8 pg/mL.

### Immunofluorescence and Imaging

48 h following MOCK or ZIKV infection, neuronal cultures were washed twice in Phosphate-buffered saline (PBS) and fixed with 4% formaldehyde in PBS for 30 min. After fixation, cells were washed three times with PBS and permeabilized in PBS containing 0.3% Triton, for 20 min. Primary antibodies were diluted in permeabilization solution as follows: mouse anti-4G2 (1:300), mouse anti-NeuN (1:500), rabbit anti-active caspase 3 (1:500), and rabbit anti-Iba1 (1:500) and incubated overnight at 4°C. Cells were washed three times with PBS and incubated with goat anti-mouse conjugated with Alexa Fluor 546 and goat anti-rabbit conjugated with Alexa Fluor 488 antibodies (diluted 1:500 in permeabilization solution) for 60 min. NucBlue™ fixed cell stain was used to label nuclei with DAPI. Image acquisition was performed using a Zeiss LSM 880 confocal system equipped with a 40×/1.30 oil DIC M27 objective. Zen 2 software was used to adjust the settings for wavelength detection of immunolabeled proteins as follows: DAPI was imaged by detection between 410 and 496 nm, Alexa Fluor 488 and Alexa Fluor-labeled antibodies were detected between 499–555 and 560–679 nm, respectively. Sequential excitation of fluorophores was performed using 405, 488, and 532 nm lasers for DAPI, Alexa Fluor 488, and Alexa Fluor 546, respectively.

### Quantitative RT-qPCR

RNA was isolated using TRIzol™ reagent as per the manufacturer’s instructions (Thermo Scientific). RNA was resuspended in 15 µL of nuclease-free water, and its concentration was analyzed by spectrophotometer (NanoDrop™, Thermo Scientific). cDNAs were prepared from 2 µg of total RNA extracted in a 20-µL final reverse transcription reaction. RT-qPCR was performed from 10× diluted cDNA and using Power SYBR™ Green PCR Master Mix in the QuantStudio™ 7 Flex real-time PCR system platform (Applied Biosystems). All RT-qPCR assays were performed to detect viral RNA and quantify mRNA levels of the following genes: mus musculus tumor necrosis factor (*Tnf-α*); mus musculus interleukin 1 beta (*Il1β*); mus musculus glutaminase (*Gls*); mus musculus solute carrier family 1 (glial high-affinity glutamate transporter) (*Slc1a2*); mus musculus glutamate receptor, ionotropic, NMDA2B (*Grin2b*); ZIKV RNA—genome reference available at GenBank: KX197192.1; and mus musculus ribosomal protein L32 (*Rpl32*). Primers were designed using Primer3Plus Program (26): *Tnfα* (forward: 5′-GCTGAGCTCAAACCCTGGTA-3′; reverse: 5′-CGGACTCCGCAAAGTCTAAG-3′); *Il1β* (forward: 5′-GGGCCTCAAAGGAAAGAATC-3′; reverse: 5′-TACCAGTTGGGGAACTCTGC-3′); *Gls* (forward: 5′-GGCAAAGGCATTCTATTGGA-3′; reverse: 5′-TTGGCTCCTTCCCAACATAG-3′); *Slc1a2* (forward: 5′-ATTGGTGCAGCCAGTATTCC-3′; reverse: 5′-CCAGCTCAGACTTGGAAAGG-3′); *Grin2b* (forward: 5′-GTGAGAGCTCCTTTGCCAAC-3′; reverse: 5′-ATGAAAGGGTTTTGCGTGAC-3′); ZIKV (forward: 5′-TCAAACGAATGGCAGTCAGTG-3′; reverse: 5′-GCTTGTTGAAGTGGTGGGAG-3′); and *Rpl32* (forward: 5′-GCTGCCATCTGTTTTACGG-3′; reverse: 5′-TGACTGGTGCCTGATGAACT-3′). Previous verification of undesired secondary formations or dimers between primers were performed using “OligoAnalyser 3.1” tool (Integrated DNA Technologies©), available at https://www.idtdna.com/calc/analyzer. All primers used in this work were validated by serial dilution assay and the reaction efficiency was calculated, comprising 90–110% (data not shown). All RT-qPCRs showed good quality of amplification and changes in gene expression were determined with the 2^−ΔCt^ method using *Rpl32* for normalization.

### Data Analysis

Means ± SEM are shown for the number of independent experiments indicated in *Figure Legends*. GraphPad Prism™ software was used to analyze data for statistical significance determined by either unpaired *t*-test (for comparing two groups) or one-way or two-way analysis of variance testing followed by Bonferroni *post hoc* multiple comparison testing.

## Results

To investigate the mechanism underlying ZIKV-induced neuronal cell death, we employed primary neuronal cell cultures from the corticostriatal region of mouse embryo brains. Over 99.5% (1,030 out of 1,035) of these cells consisted of neurons, as they were positive for the neuronal marker NeuN (Figure S1A in Supplementary Material). Only 5 out of 846 of the analyzed cells (0.5 ± 0.22%) were positively labeled for Iba1 (Figure S1B in Supplementary Material), a microglia marker. Moreover, ZIKV infection did not increase the number of microglia cells (0.5 ± 0.23%) and no microglia infected with ZIKV was found in these cultures. There was no cell positive for glial fibrillary acidic protein (Figure S1C in Supplementary Material), an astrocyte marker. Therefore, the primary cell cultures employed here can be regarded as pure neuronal cultures.

**Figure 1:**
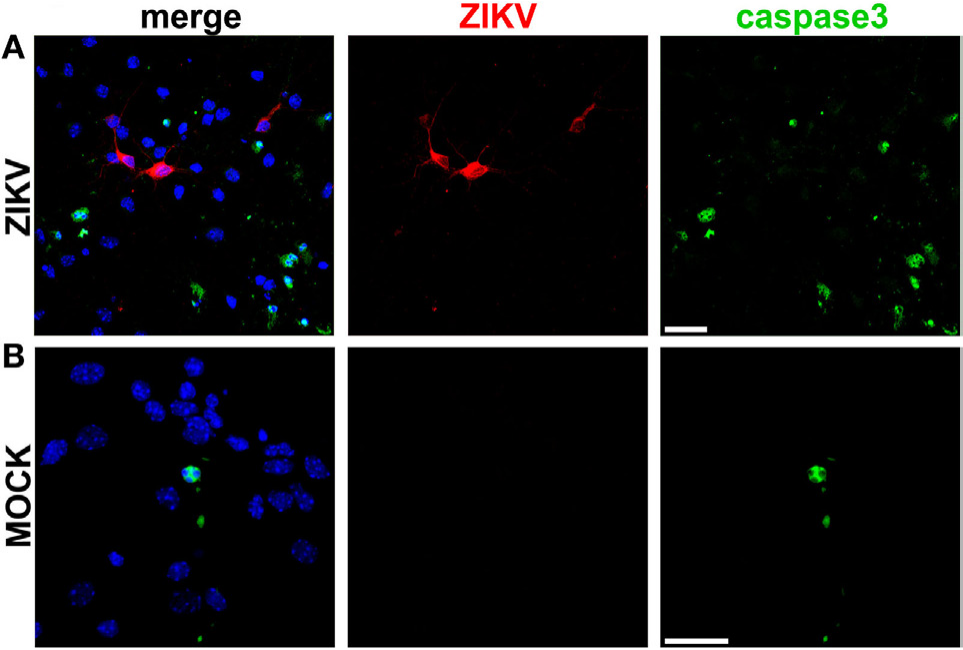
Zika virus (ZIKV) induces apoptosis in a non-cell autonomous manner. Shown are representative laser scanning confocal micrographs from primary cultured corticostriatal neurons infected with ZIKV **(A)** or MOCK-infected **(B)** for 48 h. Immunofluorescence labeling was performed using anti-ZIKV (red) and anti-active caspase 3 (green) antibodies. Cells nuclei are labeled with DAPI (blue). Panels on the left show merged image of all three fluorescent markers. Size bar corresponds to 20 µm in all images.

Primary neuronal cultures were then infected with a Brazilian isolate of ZIKV, ZIKV HS-2015-BA-01 strain. Control cultures were MOCK-infected using supernatant cultured medium from a suspension of mosquito C6/36 *A. Albopictus*-cultured cells. Forty-eight hours following infection, primary neurons were immunolabeled using specific antibodies for either ZIKV or active caspase 3. Approximately one third (28.0 ± 2.35%) of the cells present in the ZIKV-infected cultures were positive for the active form of caspase 3 (Figure 1A), compared to only 11.51 ± 1.43% of the cells in the MOCK-infected culture (Figure 1B), indicating that ZIKV infection triggers apoptosis. Interestingly, although several neurons were positive for ZIKV, we identified only 1 cell positive for ZIKV among the 228 cells positive for caspase 3. In fact, most ZIKV-positive neurons were surrounded by caspase 3-labeled neurons that appeared not to be infected by the virus (Figure 1A). These data indicate that ZIKV might trigger apoptosis mostly in a non-cell autonomous manner. Thus, we hypothesize that ZIKV-infected neurons may release pro-apoptotic factors that could trigger cell death of nearby neuronal cells.

**Figure 2:**
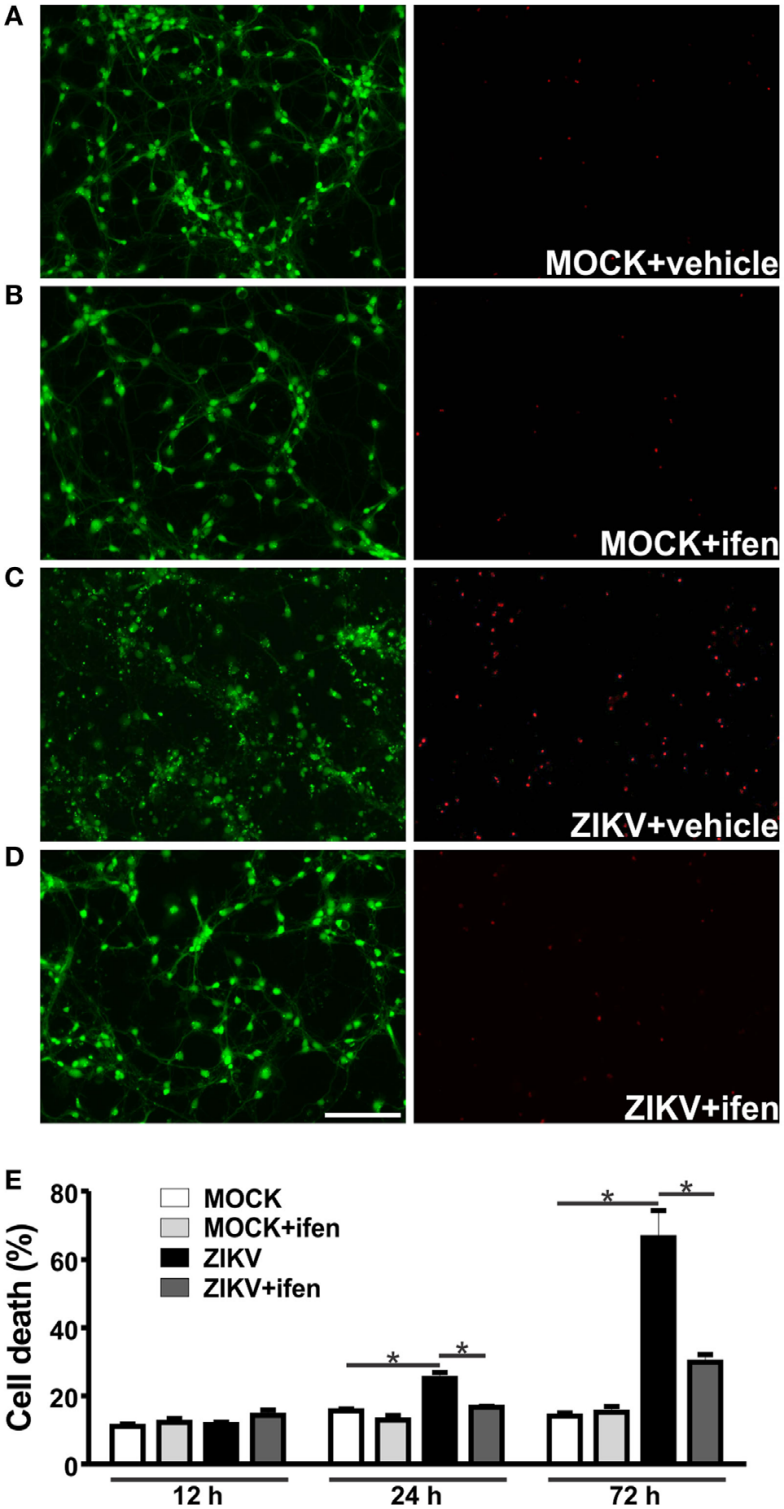
Ifenprodil rescues Zika virus (ZIKV)-induced neurotoxicity. Shown are representative images for primary cultured corticostriatal neurons labeled with calcein AM (green, live cells) and ethidium homodimer-1 (red, dead cells) that were MOCK-infected and either untreated (vehicle) **(A)** or treated with 0.01 µM ifenprodil **(B)** or that were ZIKV-infected and either untreated **(C)** or treated with 0.01 µM ifenprodil **(D)**. Size bar corresponds to 50 µm in all images. **(E)** Graph shows percentage of neuronal cell death in primary cultured corticostriatal neurons that were either untreated or treated with ifenprodil 0.01 µM for 12, 24, or 72 h following MOCK or ZIKV infection. Data represent the means ± SEM, *n* = 4. * indicates significant differences (*p* < 0.05).

We have previously demonstrated that ZIKV leads to high levels of neuronal cell death (22). Corroborating our previous results, ZIKV-infected neurons exhibited high levels of neuronal cell death 72 h following virus infection, as compared to that of MOCK-infected cells (Figure 2). To investigate early events that could be responsible for ZIKV-mediated neuronal cell death, we analyzed neurons at 12 and 24 h following virus infection. High levels of ZIKV RNA were present at 12 and 24 h following virus infection (Figure S2 in Supplementary Material). However, neuronal cell death levels higher than that of MOCK-infected neurons could only be observed at 24 h after ZIKV infection (Figure 2E). Therefore, 12 h following ZIKV infection represents a time point without overt neuronal cell death. We have recently demonstrated that NMDAR blockade abrogates ZIKV-induced neuronal cell death (22). In agreement with these results, GluN2B containing NMDAR blockade by 0.01 µM ifenprodil abolished ZIKV-triggered neuronal cell death at 24 and 72 h following virus infection (Figure 2).

**Figure 3:**
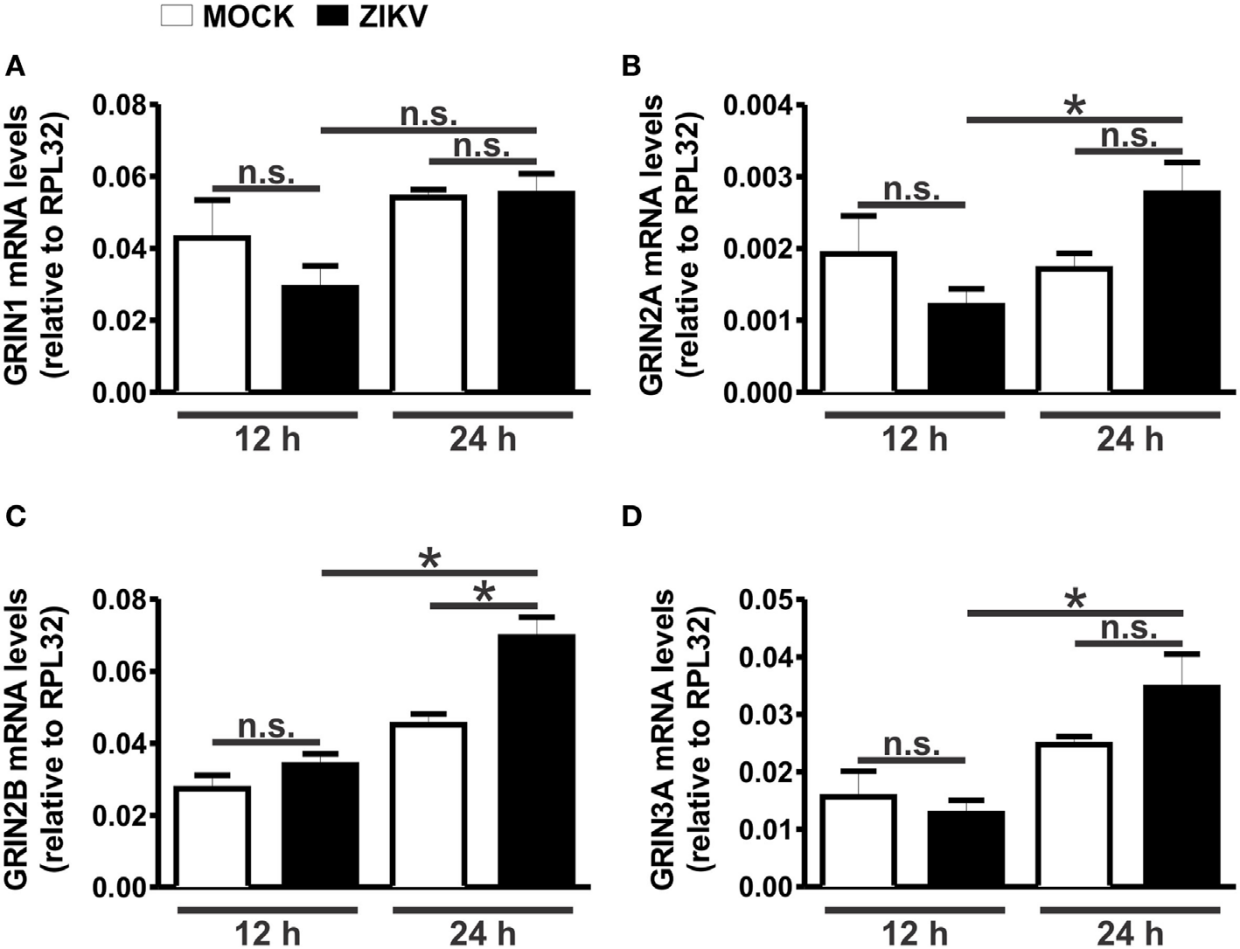
Zika virus (ZIKV) infection increases GluN2 expression. Graphs show mRNA levels of the *N*-methyl-d-aspartate receptor subunits, GluN1 (GRIN1) **(A)**, GluN2A (GRIN2A) **(B)**, GluN2B (GRIN2B) **(C)**, and GluN3A (GRIN3A) **(D)**, in primary cultured corticostriatal neurons, 12 and 24 h following MOCK or ZIKV infection. mRNA levels were assessed by quantitative RT-PCR, which was performed in triplicate and normalized to RPL32 mRNA levels. Data represent the means ± SEM, *n* = 6. n.s. indicates not significant and * indicates significant difference (*p* < 0.05).

To further explore the role of NMDARs in ZIKV-mediated neuronal cell death, we first evaluated the expression levels of different subunits of NMDARs at 12 and 24 h following virus infection. NMDARs are heterotetramers consisting of two obligatory GluN1 subunits and two additional GluN2 or GluN3 subunits (27). ZIKV infection did not alter the levels of GluN1 (Figure 3A), GluN2A (Figure 3B), and GluN3A (Figure 3D) mRNAs, as compared to MOCK-infected cultures. Although a strong tendency toward an increase in the expression of GluN2A (Figure 3B) and GluN3A (Figure 3D) was observed 24 h after ZIKV infection, no significant statistical difference was found when comparing to MOCK-infected controls. In addition, GluN2B expression levels were not increased 12 h following ZIKV infection, as compared to MOCK-infected controls (Figure 3C). However, ZIKV-infected cultures exhibited increased expression of GluN2A (Figure 3B), GluN2B (Figure 3C) and GluN3A (Figure 3D) at 24 h following infection, as compared to that of 12 h following virus infection. Moreover, at 24 h following ZIKV infection, GluN2B mRNA levels were significantly increased, as compared to that of MOCK (Figure 3C). These data further support the concept that blockade of GluN2B-containing NMDARs may offer a good therapeutic strategy to prevent ZIKV-mediated neuronal cell death.

**Figure 4:**
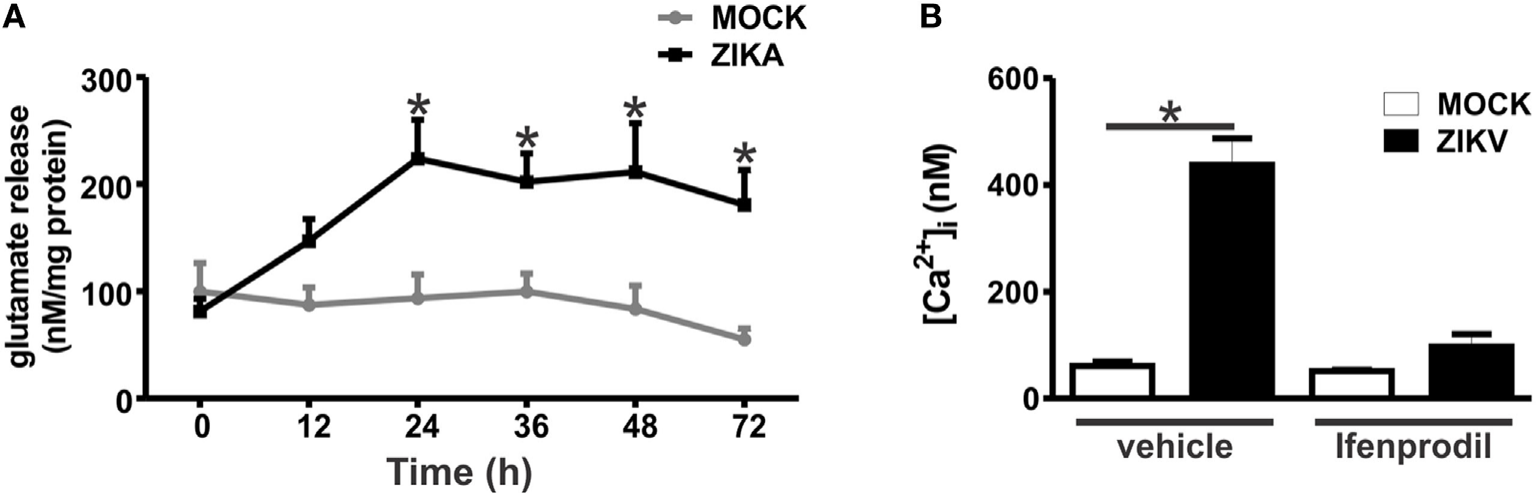
Zika virus (ZIKV) infection induces increased levels of extracellular glutamate and augmented Ca^2+^ flux through GluN2B-containing *N*-methyl-d-aspartate receptors. **(A)** Graph shows a time course of the concentration levels of extracellular glutamate in primary cultured corticostriatal neurons that were either MOCK-infected or infected with ZIKV. Data represent the means ± SEM, *n* = 6–10. * indicates significant difference as compared to MOCK-infected neurons (*p* < 0.05). **(B)** Graph shows intracellular Ca^2+^ concentration ([Ca^2+^]_i_) levels in primary cultured corticostriatal neurons that were either untreated (vehicle) or treated with 0.01 µM ifenprodil for 48 h following MOCK or ZIKV infection. Data represent the means ± SEM, *n* = 4–8. * indicates significant difference (*p* < 0.05).

Our next step was to determine whether glutamate levels were increased in ZIKV-infected cultures, which could contribute to NMDAR overactivation and, thus, neuronal cell death. Glutamate levels were increased in ZIKV-infected cultures, as compared to that of MOCK-treated cultures (Figure 4A). Although glutamate levels appeared already higher at 12 h following ZIKV infection, a significant difference was only observed at 24 h and at later time points (Figure 4A). Therefore, increased glutamate levels could account for increased NMDAR activation. Overactivation of NMDARs can lead to excitotoxicity due to increased intracellular Ca^2+^ entry into the cell (28). As GluN2B expression and glutamate levels were increased due to ZIKV infection, we investigated whether Ca^2+^ levels were also augmented in ZIKV-infected neuronal cultures. Neuronal cultures infected with ZIKV exhibited high levels of intracellular Ca^2+^ when compared to MOCK-infected cultures (Figure 4B). Moreover, treatment with 0.01 µM ifenprodil was efficient to completely rescue this increase in intracellular Ca^2+^ levels exhibited by ZIKV-infected cultures (Figure 4B), indicating that GluN2B-containing NMDARs are the main channels responsible for the increase in Ca^2+^ entry triggered by ZIKV infection.

**Figure 5:**
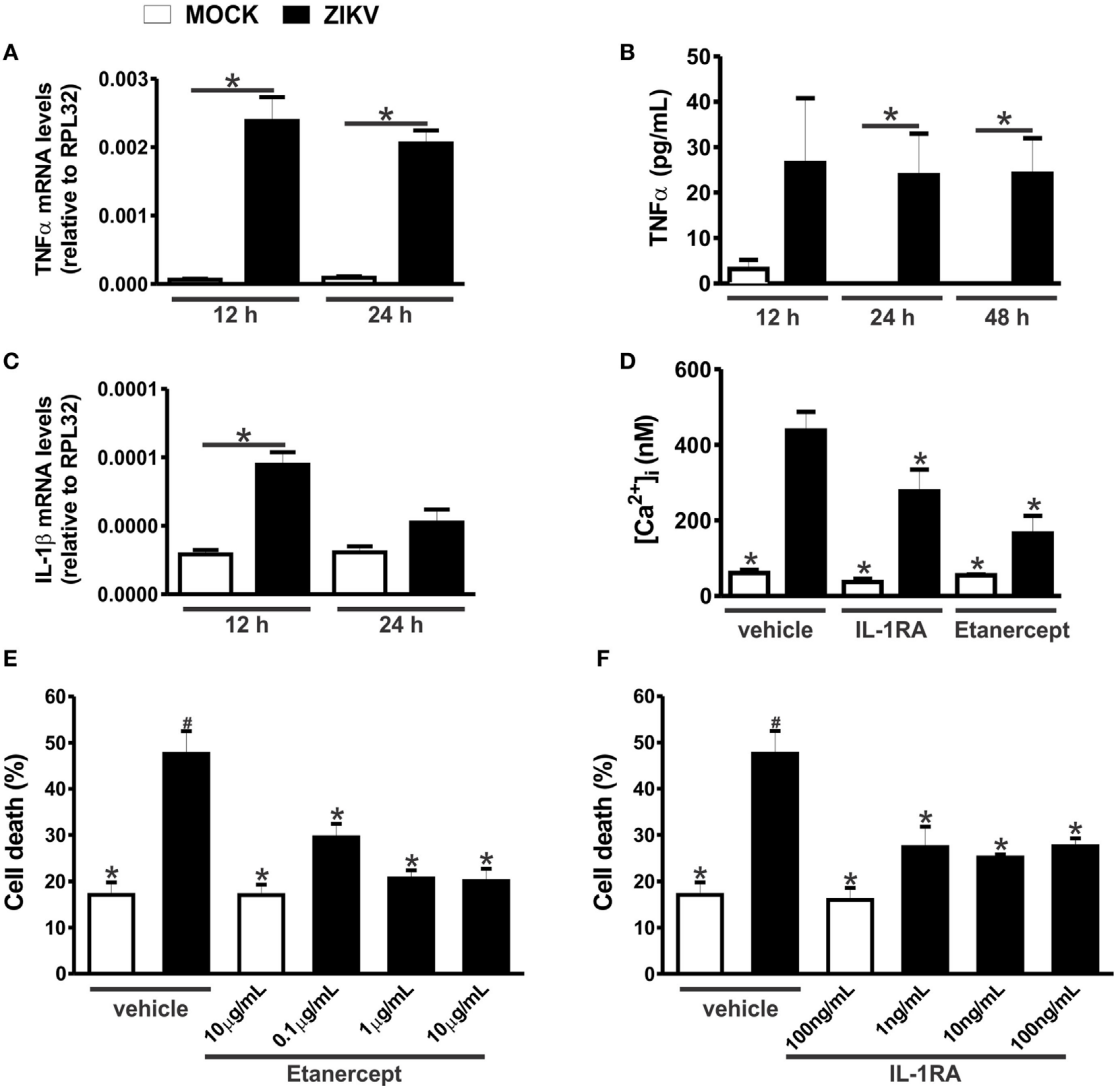
Zika virus (ZIKV) infection induces tumor necrosis factor-α (TNF-α) and interleukin-1β (IL-1β) expression, facilitating excitotoxicity. Graphs show mRNA **(A)** and protein **(B)** levels of TNF-α and mRNA levels of IL-1β **(C)**, in primary cultured corticostriatal neurons, 12 and 24 h following MOCK or ZIKV infection. mRNA levels were assessed by quantitative RT-PCR, which was performed in triplicate and normalized to RPL32 mRNA levels, and protein levels were assessed by ELISA assay. Data represent the means ± SEM, *n* = 6–8. * indicates significant differences (*p* < 0.05). **(D)** Graph shows intracellular Ca^2+^ concentration ([Ca^2+^]_i_) levels in primary cultured corticostriatal neurons that were either untreated (vehicle) or treated with 10 ng/mL IL-1RA or 1 µg/mL etanercept for 48 h following MOCK or ZIKV infection. Data represent the means ± SEM, *n* = 5–8. * indicates significant differences as compared to ZIKV infected neurons (*p* < 0.05). Graphs show percentage of neuronal cell death in primary cultured corticostriatal neurons that were either untreated (vehicle) or treated with IL-1RA 1, 10, or 100 ng/mL **(E)** or etanercept 0.1, 1, or 10 µg/mL **(F)** for 48 h following MOCK or ZIKV infection. Data represent the means ± SEM, *n* = 4–5. * indicates significant differences as compared to ZIKV-infected neurons and ^#^ indicates significant differences as compared to MOCK-infected neurons (*p* < 0.05).

Tumor necrosis factor-α can increase the expression of glutaminase, the enzyme responsible for glutamate synthesis (29, 30). Moreover, both IL-1β and TNF-α can decrease the reuptake of glutamate, thus increasing the extracellular levels of glutamate (31, 32). In addition, both TNF-α and IL-1β can sensitize NMDARs, increasing Ca^2+^ entry and, thus, excitotoxicity (33, 34). In this context, we decided to investigate whether the levels of TNF-α and IL-1β were increased in ZIKV-infected neuronal primary cultures. TNF-α mRNA levels were increased in neurons infected for 12 and 24 h with ZIKV, as compared to that of MOCK-infected cultures (Figure 5A). TNF-α protein levels were also elevated in the supernatant of cultures infected with ZIKV for 24 and 48 h, as compared to that of MOCK cultures (Figure 5B). In addition, IL-1β mRNA levels were increased 12 h after virus infection (Figure 5C). However, IL-1β protein levels were not detected by ELISA. Our next step was to determine whether this modest neuronal-derived increase in IL-1β and TNF-α production was sufficient to facilitate neuronal cell death. To address that, TNF-α and IL-1β receptor were blocked with etarnecept and IL-1RA, respectively, and ZIKV-induced neuronal cell death was measured. Etarnecept treatment at all tested concentrations (0.1, 1, and 10 µg/mL) led to a decrease in ZIKV-induced neuronal cell death (Figure 5E). IL-1β receptor blockade by IL-1RA also decreased neuronal cell death at all tested concentrations (1, 10, and 100 ng/mL) (Figure 5F). Importantly, neuronal cell death levels of ZIKV-infected cultures in the presence of either etarnecept (Figure 5E) or IL-1RA (Figure 5F) were not different than that of MOCK-infected cultures. These data clearly demonstrate the importance of these inflammatory cytokines in the neuronal cell death mechanism triggered by ZIKV. Next, we tested whether TNF-α and IL-1β could contribute to increased glutamate levels by increasing glutaminase and decreasing glutamate transporter (SLC1a) mRNA levels in neurons. No statistical difference was observed when comparing MOCK- and ZIKV-infected cultures in terms of glutaminase expression (Figure S3A in Supplementary Material). Contrary to our hypothesis, SLC1a expression levels were increased in neuronal cultures 24 h after ZIKV infection, what could constitute a protective mechanism to avoid further increases in extracellular glutamate levels (Figure S3B in Supplementary Material). Our next step was to investigate whether TNF-α and IL-1β could be facilitating NMDAR sensitization. ZIKV infection enhanced intracellular Ca^2+^ levels and this enhancement was completely blocked by ifenprodil, indicating that increased intracellular Ca^2+^ levels was primarily due to Ca^2+^ flux through GluN2B-containing NMDARs (Figure 4B). To determine whether TNF-α and IL-1β were contributing for NMDAR sensitization, we blocked TNF-α and IL-1β receptor using 1 µg/mL etanercept and 10 ng/mL IL-1RA, respectively, and evaluated intracellular Ca^2+^ levels. In agreement with our hypothesis, either TNF-α or IL-1β blockade was efficient to decrease intracellular Ca^2+^ levels (Figure 5D). These data suggest that TNF-α and IL-1β production triggered by ZIKV was effective to sensitize NMDARs, leading to increased Ca^2+^ entry and excitotoxicity.

**Figure 6:**
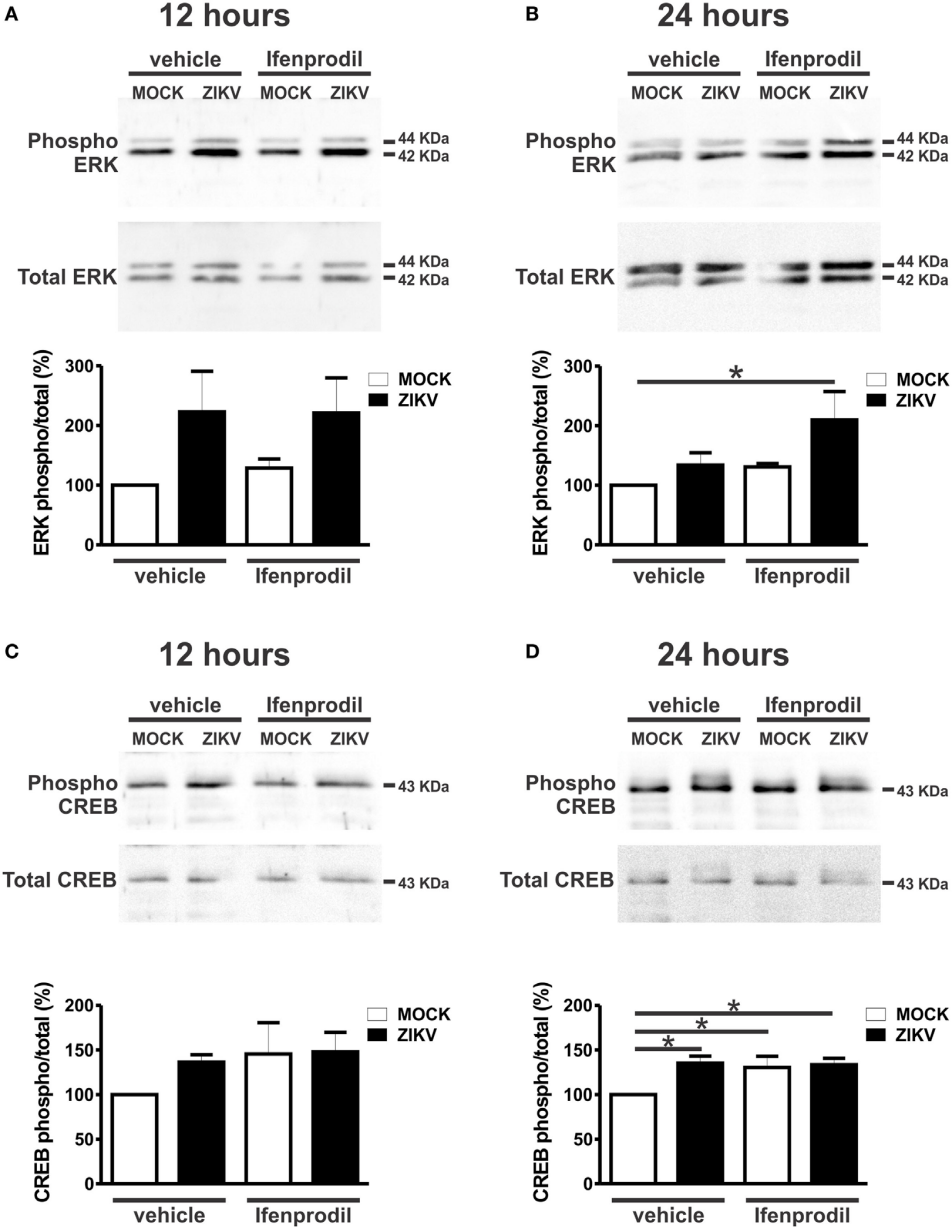
Ifenprodil treatment of Zika virus (ZIKV)-infected cultures lead to increased extracellular signal-regulated kinase (ERK) and cAMP response element-binding protein (CREB) phosphorylation. Shown are representative immunoblots for phospho- (upper panel) and total-ERK expression (lower panel) and graphs depicting the densitometric analysis of phospho-ERK normalized to total-ERK expression in primary cultured corticostriatal neurons that were either untreated (vehicle) or treated with 0.01 µM ifenprodil for 12 **(A)** or 24 **(B)** hours following MOCK or ZIKV infection. 100 µg of cell lysate was used for each sample. Data represent the means ± SEM, *n* = 5–6. * indicates significant difference (*p* < 0.05). Shown are representative immunoblots for phospho- (upper panel) and total CREB expression (lower panel) and graphs depicting the densitometric analysis of phospho-CREB normalized to total CREB expression in primary cultured corticostriatal neurons that were either untreated (vehicle) or treated with 0.01 µM ifenprodil for 12 **(C)** or 24 **(D)** hours following MOCK or ZIKV infection. 100 µg of cell lysate was used for each sample. Data represent the means ± SEM, *n* = 4–6. * indicates significant differences (*p* < 0.05).

To further understand the neuroprotective mechanism underlying GluN2B-containing NMDARs blockade, we investigated which cell survival signaling pathways were activated by ZIKV infection. Ifenprodil normalized the increased levels of intracellular Ca^2+^ triggered by ZIKV, which could greatly contribute to neuroprotection (Figure 4B). However, the neuroprotective pathway comprising ERK and CREB can be stimulated by glutamate receptors other than GluN2B-containing NMDARs (35). For instance, as ifenprodil only blocks GluN2B-containing NMDARs, glutamate could still activate GluN2A-containing NMDARs, which are predominantly synaptic NMDARs that were shown to promote neuronal survival *via* CREB/brain-derived neurotrophic factor (BDNF) signaling (36). As ZIKV-infected cultures exhibited high levels of extracellular glutamate (Figure 4A), we decided to investigate whether the levels of phosphorylation/activation of ERK and CREB were altered by ZIKV infection and 0.01 µM ifenprodil treatment. No difference was observed when comparing the activation of ERK and CREB in MOCK- and ZIKV-infected cultures at 12 h after virus infection (Figures 6A,C). However, 24 h following infection, ERK activation was increased in neurons that were infected with ZIKV and treated with ifenprodil, as compared to that of MOCK-infected cultures (Figure 6B). ZIKV infection, in the presence or absence of ifenprodil treatment, increased CREB phosphorylation, as compared to MOCK (Figure 6D). Moreover, ifenprodil treatment in the absence of ZIKV was also efficient to activate CREB (Figure 6D). CREB can be activated *via* NMDARs through cell signaling pathways other than ERK, which could account for the ERK-independent activation of CREB observed in our experiments (37). Altogether, these data indicate that the cell survival signaling pathway downstream from NMDARs ERK/CREB is activated following ZIKV infection and GluN2B-containing NMDAR blockade.

## Discussion

### ZIKV Induces Neuronal Apoptosis in a Non-Cell Autonomous Manner

Several flaviviruses, including Saint Louis encephalitis virus (38), Japanese encephalitis virus (JEV) (39, 40), and West Nile virus (WNV) (41), have been shown to induce neuronal apoptosis, an effect that appears to contribute to the neurological damage caused by these viruses. Virus-triggered neuronal apoptosis can be immune-mediated or induced by virus cell autonomous injury. For instance, flaviviruses can promote neuronal injury by altering the expression of pro- and anti-apoptotic proteins, thus, facilitating death of infected cells (39, 40, 42). It has been shown that primary cultured cortical neurons infected with WNV undergo apoptosis and that 96% of cells that were caspase 3 positive were also positive for WNV (41). These results are different from the data obtained in our study, as the great majority of neurons that were positive for caspase 3 were negative for ZIKV. Thus, the cell response triggered by ZIKV might differ from other flaviviruses. Previous studies have demonstrated that ZIKV induces apoptosis of human neural progenitor cells (19, 20, 43–45). Although the number of cells that were positive for both caspase 3 and ZIKV was not determined, it is possible to notice that several cells that were positive for ZIKV were not positive for caspase 3 in these studies (43, 45). Another study has shown that brain slices obtained from mice subjected to intracranial injection of ZIKV exhibits only a few cells positive for both caspase 3 and ZIKV (44), which corroborates our data showing that ZIKV induces apoptosis mostly through a non-cell autonomous mechanism. In this context, ZIKV will be able to efficiently replicate in infected neurons, increasing viral load, and otherwise healthy neurons will undergo apoptosis, exacerbating neurodegeneration. However, at this point, it is still unclear why ZIKV infected cells could be protected from apoptosis. It has been demonstrated that ZIKV infection upregulates PRPF8, which is a splicing factor known to have an anti-apoptotic effect in neurons infected with Picornavirus (46). Thus, ZIKV could protect infected neurons by altering the expression of apoptotic factors.

### ZIKV Induction of TNF-α and IL-1β Production by Neurons

Most encephalitic viruses induce production of inflammatory factors by glial cells. For example, JEV can infect both neuronal and glial cells, promoting the release of inflammatory factors, including TNFα and IL-1β, and glutamate by microglia (30). ZIKV is able to infect microglia as the virus was detected in microglia obtained from human fetal brain tissue (47). Importantly, ZIKV-infected microglia exhibits increased expression levels of several chemokines and cytokines, including IL-6, TNF-α, IL-1β, and monocyte chemotactic protein 1 (47). We have identified TNF-α and IL-1β as important factors triggering ZIKV-induced neuronal cell death. Interestingly, even though ZIKV induced only a mild increase in TNF-α and IL-1β production by neuronal cultures, this increase was enough to sensitize NMDARs and facilitate neuronal cell death. Although astrocytes and neurons can produce TNF-α, the major source of this cytokine is microglia. However, in certain specific situations, neurons can also increase TNF-α production (48, 49). For example, WNV induces the expression of IL-1β, IL-6, IL-8, and TNF-α by neuronal cells and these neuron-derived cytokines can mediate the activation of astrocytes and contribute to WNV-induced neurotoxicity (50). Thus, it is possible that ZIKV infection could activate neuronal production of TNFα and IL-1β. We also have to consider that the microglia present in our neuronal cultures might contribute to the production of these cytokines. However, this seems rather unlikely as the number of microglia was very small (~0.5%) and was not increased due to ZIKV infection. Future studies will be important to determine the role of different brain cell types in ZIKV-induced neuroinflammation.

### TNF-α and IL-β Sensitize NMDAR and Trigger Neuronal Cell Death

**Figure 7:**
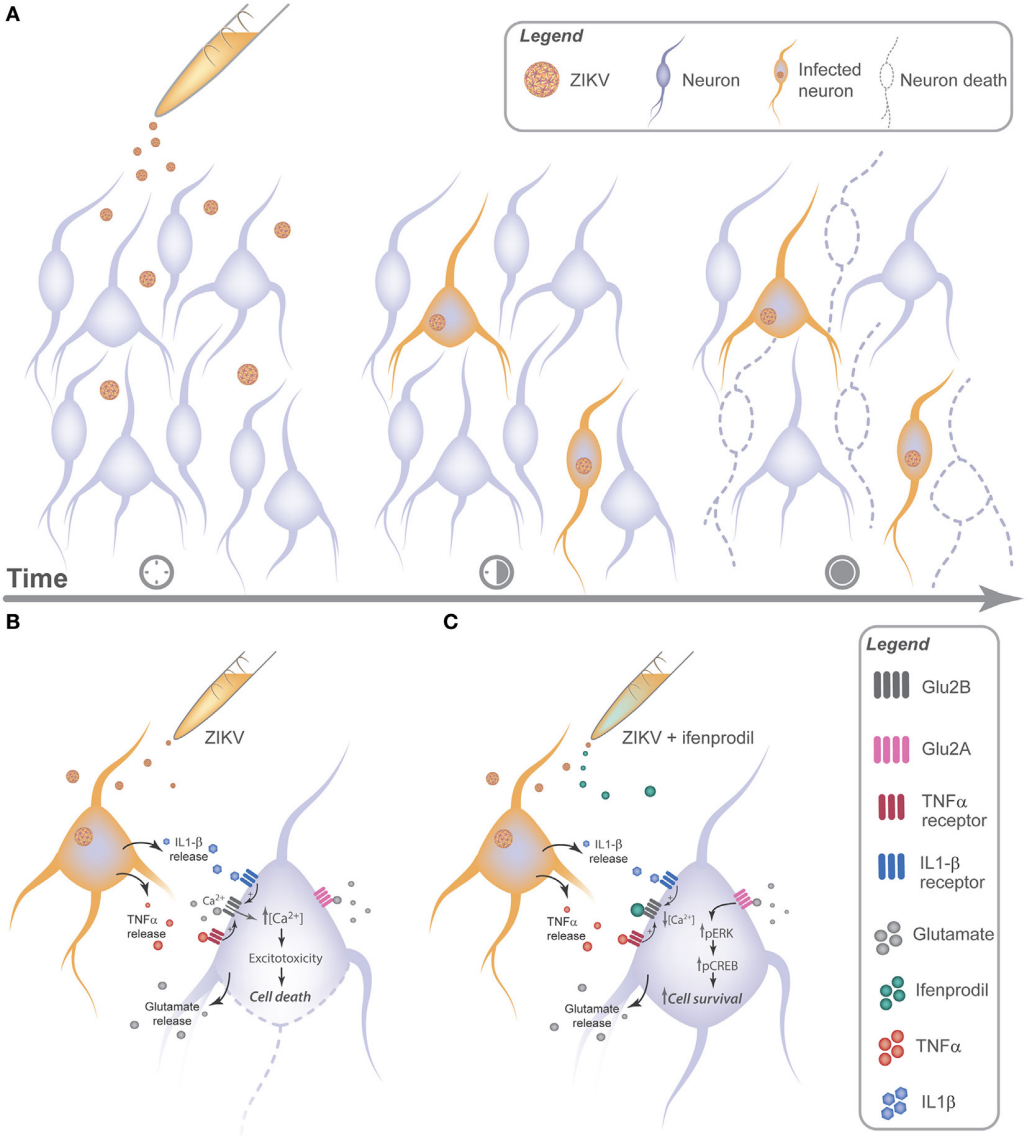
Zika virus (ZIKV) promotes neuronal cell death *via* GluN2B activation in a non-cell autonomous manner by releasing neurotoxic factors. **(A)** ZIKV can be detected as early as 12 h following viral infection. 24 h later, cell injury can be observed in uninfected neurons nearby infected cells, indicating a cross talk between infected and uninfected neurons. **(B)** Neuronal cultures infected with ZIKV release glutamate and cytokines, triggering GluN2B-containing *N*-methyl-d-aspartate receptor (NMDAR) sensitization and increasing the influx of extracellular Ca^2+^, which facilitates excitotoxicity. **(C)** Ifenprodil treatment of ZIKV-infected cultures reduces viral-mediated increased Ca^2+^ flux through GluN2B-containing NMDARs. Activation of GluN2A-containing NMDARs by glutamate in the presence of ifenprodil leads to ERK and CREB activation, which can increase the expression of genes important for neuronal survival.

Our results show that TNF-α and IL-1β appeared to be key players of ZIKV-induced neuronal cell death. It has been previously demonstrated that these inflammatory cytokines potentiate excitotoxicity (51). For instance, IL-1β can increase Ca^2+^ flux through NMDARs (34). The mechanism underlying NMDAR sensitization by IL-1β involves the phosphorylation of NMDAR subunits GluN2A/B by Src tyrosine kinase (34). TNF-α can also sensitize NMDARs, increasing NMDAR-dependent Ca^2+^ entry and facilitating neurotoxicity (33). Moreover, it has also been shown that TNF-α can increase the localization of ionotropic glutamate receptors, including NMDARs, to synapses (52). Our results demonstrate that ZIKV infection augments Ca^2+^ flux through NMDARs, as GluN2B-containing NMDAR blockade by ifenprodil completely abolished the increase in intracellular Ca^2+^ levels. Importantly, ZIKV infection increased IL-1β and TNF-α expression levels and blockade of these cytokines decreased Ca^2+^ influx and neuronal cell death. These results strongly indicate that IL-1β and TNF-α are sensitizing NMDARs and facilitating ZIKV-induced excitotoxicity. Damaged or dying brain cells release high levels of glutamate (53), which could underlie the increase in glutamate levels and contribute for neuronal cell death propagation in a non-cell autonomous way (Figure 7).

We show here that GluN2B expression is increased in cultures infected with ZIKV. At this point, it is unclear why the expression of this NMDAR subunit is augmented. However, it has been demonstrated that TNF-α increases GluN1 expression (54) and that IL-1β increases de expression of GluN2A/B (55). Thus, it is possible that IL-1β could contribute to the observed increased expression of GluN2B. Although so far it is not clear what is the mechanism contributing for ZIKV-mediated increase in GluN2B expression, our data clearly show that GluN2B-containing NMDAR is a relevant pharmacological target to treat ZIKV-mediated neurotoxicity.

### NMDAR Dual Role in Cell Survival and Neurodegeneration

Our results clearly demonstrate that blockade of GluN2B-containing NMDARs by ifenprodil leads to neuroprotection against ZIKV infection. Numerous studies indicate that GluN2A mediates the protective pathway and GluN2B contributes to the excitotoxic pathway (27, 56–59). NMDARs can be localized at either the synaptic or extrasynaptic region, and a few years ago, it was proposed that signaling resulting from synaptic and extrasynaptic NMDAR stimulation is linked to neuronal survival and death, respectively (36). It is generally thought that most GluN2A-containing NMDARs are synaptic whereas GluN2B-containing NMDARs are extrasynaptic (59–63). However, synaptic/extrasynaptic distribution of NMDARs is not a clear-cut spatial demarcation, as GluN2A subunits have been found at extrasynaptic sites and GluN2B at the synaptic region (27, 64, 65). Synaptic NMDARs are in general transiently and intensely activated by the trans-synaptic release of glutamate, while extrasynaptic NMDARs are more commonly activated by chronic exposure to high levels of glutamate. Importantly, our results indicated that ZIKV infection led to sustained high levels of extracellular glutamate in the culture, which could underlie the activation of extrasynaptic NMDARs. Corroborating this hypothesis, ifenprodil rescued ZIKV-induced neuronal cell death.

Ca^2+^ influx evoked by the activation of synaptic NMDAR is well tolerated by neurons and can trigger the activation of CREB, which increases the expression of genes important for neuronal survival, including BDNF (36, 57, 66). In sharp contrast, comparable Ca^2+^ transients induced by activation of extrasynaptic NMDARs trigger a CREB shut-off pathway and mitochondrial membrane potential dysfunction, leading to neuronal cell death (36). In addition to BDNF, CREB targets genes that are important for reducing apoptosis by rendering mitochondria more resistant to cellular stress and toxic insults (67–70). Activation of CREB by NMDARs can occur *via* ERK and synaptic NMDAR stimulation promotes sustained ERK activity, whereas activation of all NMDARs by bath application of glutamate results in ERK activation that is followed by rapid inactivation (71–75). In agreement with these studies, we did not observe ERK activation when cultures were infected with ZIKV and glutamate levels were high, although an increase in ERK activation was observed when ifenprodil was added to ZIKV-infected cultures. Therefore, it is possible that GluN2B-containing NMDAR blockade decreased ERK inactivation allowing this kinase to phosphorylate its downstream targets, including CREB. However, we observed activation of CREB in ZIKV-infected cultures, regardless of ifenprodil treatment. This is probably because CREB can be activated by kinases other than ERK. For example, it has been shown that synaptic NMDAR stimulation can lead to activation of the calmodulin-dependent protein kinase IV, which promotes rapid activation of CREB, as opposed to the slower acting and long-lasting effects produced by ERK (76). Therefore, the results presented here corroborate previous studies and indicate that the increase in glutamate triggered by ZIKV may not necessarily lead to NMDAR-mediated cell death, as long as GluN2B-containing NDMARs are blocked.

## Conclusion

Zika virus induces neuronal cell death in a non-cell autonomous manner by triggering the release of cytokines, including TNF-α and IL-1β. Increased levels of TNF-α, IL-1β, and glutamate overactivates NMDARs, promoting excitotoxicity and, consequently, neuronal cell death (Figure 7). Therefore, these results help to clarify the neurotoxic mechanisms elicited by ZIKV neuronal infection.

## Ethics Statement

This study was carried out in accordance with the recommendations of the Brazilian Government (law 11794/2008a) and approved by the Committee on Animal Ethics of the UFMG (CEUA/UFMG, permit protocol no. 242/2016).

## Author Contributions

IO and FR designed the study, interpreted the data, and wrote the manuscript. IO, TC, VC, JA-S, CF, TI-T, JS, and LV performed the acquisition, analysis, and interpretation of the data. JM, AT, DS, MT, and LV substantially contributed to the conception of the work and revised it critically for important intellectual content. All the authors approved the final version of the manuscript.

## Conflict of Interest Statement

The authors declare that the research was conducted in the absence of any commercial or financial relationships that could be construed as a potential conflict of interest.

## Abbreviations

BDNF, brain-derived neurotrophic factor; CamKIV, calmodulin-dependent protein kinase IV; CREB, cAMP response element-binding protein; DIV, days *in vitro*; ERK, extracellular signal-regulated kinase; GFAP, glial fibrillary acidic protein; GLS, glutaminase; GluN, *N*-methyl d-aspartate receptor subtype; HBSS, Hank’s balanced salt solution; IL, interleukin; JEV, Japanese encephalitis virus; MCP-1, monocyte chemotactic protein 1; NeuN, neuronal marker; NMDAR, *N*-methyl-d-aspartate receptor; Rpl32, ribosomal protein L32; SLC1a, glutamate transporter; SLEV, Saint Louis encephalitis virus; TNF-α, tumor necrosis factor-α; WNV, West Nile virus; ZIKV, Zika virus.

## References

[1] Dick GW Kitchen SF Haddow AJ Zika virus. I. Isolations and serological specificity. Trans R Soc Trop Med Hyg (1952) 46:509–20. 10.1016/0035-9203(52)90042-4 12995440

[2] Chang C Ortiz K Ansari A Gershwin ME. The Zika outbreak of the 21st century. J Autoimmun (2016) 68:1–13. 10.1016/j.jaut.2016.02.006 26925496 PMC7127657

[3] Duffy MR Chen TH Hancock WT Powers AM Kool JL Lanciotti RS Zika virus outbreak on Yap Island, Federated States of Micronesia. N Engl J Med (2009) 360:2536–43. 10.1056/NEJMoa0805715 19516034

[4] Cao-Lormeau VM Roche C Teissier A Robin E Berry AL Mallet HP Zika virus, French Polynesia, South Pacific, 2013. Emerg Infect Dis (2014) 20:1085–6. 10.3201/eid2006.140138 24856001 PMC4036769

[5] Petersen E Wilson ME Touch S McCloskey B Mwaba P Bates M Rapid spread of Zika virus in the Americas – implications for public health preparedness for mass gatherings at the 2016 Brazil Olympic Games. Int J Infect Dis (2016) 44:11–5. 10.1016/j.ijid.2016.02.001 26854199

[6] de Araujo TV Rodrigues LC de Alencar Ximenes RA de Barros Miranda-Filho D Montarroyos UR de Melo AP Association between Zika virus infection and microcephaly in Brazil, January to May, 2016: preliminary report of a case-control study. Lancet Infect Dis (2016) 16:1356–63. 10.1016/S1473-3099(16)30318-8 27641777 PMC7617035

[7] Brasil P Pereira JP Jr Moreira ME Ribeiro Nogueira RM Damasceno L Wakimoto M Zika virus infection in pregnant women in Rio de Janeiro. N Engl J Med (2016) 375:2321–34. 10.1056/NEJMoa1602412 26943629 PMC5323261

[8] Cuevas EL Tong VT Rozo N Valencia D Pacheco O Gilboa SM Preliminary report of microcephaly potentially associated with Zika virus infection during pregnancy – Colombia, January–November 2016. MMWR Morb Mortal Wkly Rep (2016) 65:1409–13. 10.15585/mmwr.mm6549e1 27977645

[9] Cauchemez S Besnard M Bompard P Dub T Guillemette-Artur P Eyrolle-Guignot D Association between Zika virus and microcephaly in French Polynesia, 2013–15: a retrospective study. Lancet (2016) 387:2125–32. 10.1016/S0140-6736(16)00651-6 26993883 PMC4909533

[10] Dos Santos T Rodriguez A Almiron M Sanhueza A Ramon P de Oliveira WK Zika virus and the Guillain-Barre syndrome – case series from seven countries. N Engl J Med (2016) 375:1598–601. 10.1056/NEJMc1609015 27579558

[11] Cao-Lormeau VM Blake A Mons S Lastere S Roche C Vanhomwegen J Guillain-Barre syndrome outbreak associated with Zika virus infection in French Polynesia: a case-control study. Lancet (2016) 387:1531–9. 10.1016/S0140-6736(16)00562-6 26948433 PMC5444521

[12] Krauer F Riesen M Reveiz L Oladapo OT Martinez-Vega R Porgo TV Zika virus infection as a cause of congenital brain abnormalities and Guillain-Barre syndrome: systematic review. PLoS Med (2017) 14:e1002203 10.1371/journal.pmed.1002203 28045901 PMC5207634

[13] Gulland A Zika virus is a global public health emergency, declares WHO. BMJ (2016) 352:i657 10.1136/bmj.i657 26839247

[14] Sarno M Sacramento GA Khouri R do Rosario MS Costa F Archanjo G Zika virus infection and stillbirths: a case of hydrops fetalis, hydranencephaly and fetal demise. PLoS Negl Trop Dis (2016) 10:e0004517. 10.1371/journal.pntd.0004517 26914330 PMC4767410

[15] Calvet G Aguiar RS Melo AS Sampaio SA de Filippis I Fabri A Detection and sequencing of Zika virus from amniotic fluid of fetuses with microcephaly in Brazil: a case study. Lancet Infect Dis (2016) 16:653–60. 10.1016/S1473-3099(16)00095-5 26897108

[16] Martines RB Bhatnagar J Keating MK Silva-Flannery L Muehlenbachs A Gary J Notes from the field: evidence of Zika virus infection in brain and placental tissues from two congenitally infected newborns and two fetal losses – Brazil, 2015. MMWR Morb Mortal Wkly Rep (2016) 65:159–60. 10.15585/mmwr.mm6506e1 26890059

[17] Mlakar J Korva M Tul N Popovic M Poljsak-Prijatelj M Mraz J Zika virus associated with microcephaly. N Engl J Med (2016) 374:951–8. 10.1056/NEJMoa1600651 26862926

[18] Ventura CV Maia M Bravo-Filho V Gois AL Belfort R Jr Zika virus in Brazil and macular atrophy in a child with microcephaly. Lancet (2016) 387:228 10.1016/S0140-6736(16)00006-4 26775125

[19] Cugola FR Fernandes IR Russo FB Freitas BC Dias JL Guimaraes KP The Brazilian Zika virus strain causes birth defects in experimental models. Nature (2016) 534:267–71. 10.1038/nature18296 27279226 PMC4902174

[20] Garcez PP Loiola EC Madeiro da Costa R Higa LM Trindade P Delvecchio R Zika virus impairs growth in human neurospheres and brain organoids. Science (2016) 352:816–8. 10.1126/science.aaf6116 27064148

[21] Li C Xu D Ye Q Hong S Jiang Y Liu X Zika virus disrupts neural progenitor development and leads to microcephaly in mice. Cell Stem Cell (2016) 19:120–6. 10.1016/j.stem.2016.04.017 27179424

[22] Costa VV Del Sarto JL Rocha RF Silva FR Doria JG Olmo IG N-methyl-d-aspartate (NMDA) receptor blockade prevents neuronal death induced by Zika virus infection. MBio (2017) 8:e350–317. 10.1128/mBio.00350-17 28442607 PMC5405231

[23] Costa VV Fagundes CT Valadao DF Avila TV Cisalpino D Rocha RF Subversion of early innate antiviral responses during antibody-dependent enhancement of Dengue virus infection induces severe disease in immunocompetent mice. Med Microbiol Immunol (2014) 203:231–50. 10.1007/s00430-014-0334-5 24723052

[24] Doria JG Silva FR de Souza JM Vieira LB Carvalho TG Reis HJ Metabotropic glutamate receptor 5 positive allosteric modulators are neuroprotective in a mouse model of Huntington’s disease. Br J Pharmacol (2013) 169:909–21. 10.1111/bph.12164 23489026 PMC3687670

[25] Nicholls DG Sihra TS Sanchez-Prieto J. Calcium-dependent and -independent release of glutamate from synaptosomes monitored by continuous fluorometry. J Neurochem (1987) 49:50–7. 10.1111/j.1471-4159.1987.tb03393.x 2884279

[26] Untergasser A Nijveen H Rao X Bisseling T Geurts R Leunissen JA. Primer3Plus, an enhanced web interface to Primer3. Nucleic Acids Res (2007) 35:W71–4. 10.1093/nar/gkm306 17485472 PMC1933133

[27] Paoletti P Bellone C Zhou Q. NMDA receptor subunit diversity: impact on receptor properties, synaptic plasticity and disease. Nat Rev Neurosci (2013) 14:383–400. 10.1038/nrn3504 23686171

[28] Mody I MacDonald JF. NMDA receptor-dependent excitotoxicity: the role of intracellular Ca^2+^ release. Trends Pharmacol Sci (1995) 16:356–9. 10.1016/S0165-6147(00)89070-7 7491714

[29] Takeuchi H Jin S Wang J Zhang G Kawanokuchi J Kuno R Tumor necrosis factor-alpha induces neurotoxicity via glutamate release from hemichannels of activated microglia in an autocrine manner. J Biol Chem (2006) 281:21362–8. 10.1074/jbc.M600504200 16720574

[30] Chen CJ Ou YC Chang CY Pan HC Liao SL Chen SY Glutamate released by Japanese encephalitis virus-infected microglia involves TNF-alpha signaling and contributes to neuronal death. Glia (2012) 60:487–501. 10.1002/glia.22282 22144112

[31] Szymocha R Akaoka H Dutuit M Malcus C Didier-Bazes M Belin MF Human T-cell lymphotropic virus type 1-infected T lymphocytes impair catabolism and uptake of glutamate by astrocytes via Tax-1 and tumor necrosis factor alpha. J Virol (2000) 74:6433–41. 10.1128/JVI.74.14.6433-6441.2000 10864655 PMC112151

[32] Prow NA Irani DN. The inflammatory cytokine, interleukin-1 beta, mediates loss of astroglial glutamate transport and drives excitotoxic motor neuron injury in the spinal cord during acute viral encephalomyelitis. J Neurochem (2008) 105:1276–86. 10.1111/j.1471-4159.2008.05230.x 18194440 PMC2579753

[33] Jara JH Singh BB Floden AM Combs CK. Tumor necrosis factor alpha stimulates NMDA receptor activity in mouse cortical neurons resulting in ERK-dependent death. J Neurochem (2007) 100:1407–20. 10.1111/j.1471-4159.2006.04330.x 17241124 PMC3619402

[34] Viviani B Bartesaghi S Gardoni F Vezzani A Behrens MM Bartfai T Interleukin-1beta enhances NMDA receptor-mediated intracellular calcium increase through activation of the Src family of kinases. J Neurosci (2003) 23:8692–700. 14507968 10.1523/JNEUROSCI.23-25-08692.2003 PMC6740426

[35] Thomas GM Huganir RL MAPK cascade signalling and synaptic plasticity. Nat Rev Neurosci (2004) 5:173–83. 10.1038/nrn1346 14976517

[36] Hardingham GE Fukunaga Y Bading H. Extrasynaptic NMDARs oppose synaptic NMDARs by triggering CREB shut-off and cell death pathways. Nat Neurosci (2002) 5:405–14. 10.1038/nn835 11953750

[37] Hardingham GE Bading H. Synaptic versus extrasynaptic NMDA receptor signalling: implications for neurodegenerative disorders. Nat Rev Neurosci (2010) 11:682–96. 10.1038/nrn2911 20842175 PMC2948541

[38] Parquet MC Kumatori A Hasebe F Mathenge EG Morita K. St. Louis encephalitis virus induced pathology in cultured cells. Arch Virol (2002) 147:1105–19. 10.1007/s00705-002-0806-6 12111422

[39] Liao CL Lin YL Wang JJ Huang YL Yeh CT Ma SH Effect of enforced expression of human bcl-2 on Japanese encephalitis virus-induced apoptosis in cultured cells. J Virol (1997) 71:5963–71. 9223486 10.1128/jvi.71.8.5963-5971.1997 PMC191852

[40] Su HL Liao CL Lin YL. Japanese encephalitis virus infection initiates endoplasmic reticulum stress and an unfolded protein response. J Virol (2002) 76:4162–71. 10.1128/JVI.76.9.4162-4171.2002 11932381 PMC155064

[41] Shrestha B Gottlieb D Diamond MS. Infection and injury of neurons by West Nile encephalitis virus. J Virol (2003) 77:13203–13. 10.1128/JVI.77.24.13203-13213.2003 14645577 PMC296085

[42] Parquet MC Kumatori A Hasebe F Morita K Igarashi A. West Nile virus-induced bax-dependent apoptosis. FEBS Lett (2001) 500:17–24. 10.1016/S0014-5793(01)02573-X 11434919

[43] Xu M Lee EM Wen Z Cheng Y Huang WK Qian X Identification of small-molecule inhibitors of Zika virus infection and induced neural cell death via a drug repurposing screen. Nat Med (2016) 22:1101–7. 10.1038/nm.4184 27571349 PMC5386783

[44] Huang WC Abraham R Shim BS Choe H Page DT. Zika virus infection during the period of maximal brain growth causes microcephaly and corticospinal neuron apoptosis in wild type mice. Sci Rep (2016) 6:34793. 10.1038/srep34793 27713505 PMC5054421

[45] Tang H Hammack C Ogden SC Wen Z Qian X Li Y Zika virus infects human cortical neural progenitors and attenuates their growth. Cell Stem Cell (2016) 18:587–90. 10.1016/j.stem.2016.02.016 26952870 PMC5299540

[46] Garcez PP Nascimento JM de Vasconcelos JM Madeiro da Costa R Delvecchio R Trindade P Zika virus disrupts molecular fingerprinting of human neurospheres. Sci Rep (2017) 7:40780. 10.1038/srep40780 28112162 PMC5256095

[47] Lum FM Low DK Fan Y Tan JJ Lee B Chan JK Zika virus infects human fetal brain microglia and induces inflammation. Clin Infect Dis (2017) 64:914–20. 10.1093/cid/ciw878 28362944

[48] Ignatowski TA Noble BK Wright JR Gorfien JL Heffner RR Spengler RN. Neuronal-associated tumor necrosis factor (TNF alpha): its role in noradrenergic functioning and modification of its expression following antidepressant drug administration. J Neuroimmunol (1997) 79:84–90. 10.1016/S0165-5728(97)00107-0 9357451

[49] Liu T Clark RK McDonnell PC Young PR White RF Barone FC Tumor necrosis factor-alpha expression in ischemic neurons. Stroke (1994) 25:1481–8. 10.1161/01.STR.25.7.1481 8023366

[50] Kumar M Verma S Nerurkar VR. Pro-inflammatory cytokines derived from West Nile virus (WNV)-infected SK-N-SH cells mediate neuroinflammatory markers and neuronal death. J Neuroinflammation (2010) 7:73. 10.1186/1742-2094-7-73 21034511 PMC2984415

[51] Olmos G Llado J Tumor necrosis factor alpha: a link between neuroinflammation and excitotoxicity. Mediators Inflamm (2014) 2014:861231 10.1155/2014/861231 24966471 PMC4055424

[52] Pickering M Cumiskey D O’Connor JJ. Actions of TNF-alpha on glutamatergic synaptic transmission in the central nervous system. Exp Physiol (2005) 90:663–70. 10.1113/expphysiol.2005.030734 15944202

[53] Randall RD Thayer SA. Glutamate-induced calcium transient triggers delayed calcium overload and neurotoxicity in rat hippocampal neurons. J Neurosci (1992) 12:1882–95. 1349638 10.1523/JNEUROSCI.12-05-01882.1992 PMC6575874

[54] Weaver-Mikaere L Gunn AJ Mitchell MD Bennet L Fraser M. LPS and TNF alpha modulate AMPA/NMDA receptor subunit expression and induce PGE2 and glutamate release in preterm fetal ovine mixed glial cultures. J Neuroinflammation (2013) 10:153. 10.1186/1742-2094-10-153 24344780 PMC3878505

[55] Dong Y Kalueff AV Song C. N-methyl-d-aspartate receptor-mediated calcium overload and endoplasmic reticulum stress are involved in interleukin-1beta-induced neuronal apoptosis in rat hippocampus. J Neuroimmunol (2017) 307:7–13. 10.1016/j.jneuroim.2017.03.005 28495142

[56] Liu Y Wong TP Aarts M Rooyakkers A Liu L Lai TW NMDA receptor subunits have differential roles in mediating excitotoxic neuronal death both in vitro and in vivo. J Neurosci (2007) 27:2846–57. 10.1523/JNEUROSCI.0116-07.2007 17360906 PMC6672582

[57] Vanhoutte P Bading H. Opposing roles of synaptic and extrasynaptic NMDA receptors in neuronal calcium signalling and BDNF gene regulation. Curr Opin Neurobiol (2003) 13:366–71. 10.1016/S0959-4388(03)00073-4 12850222

[58] Monyer H Burnashev N Laurie DJ Sakmann B Seeburg PH. Developmental and regional expression in the rat brain and functional properties of four NMDA receptors. Neuron (1994) 12:529–40. 10.1016/0896-6273(94)90210-0 7512349

[59] Tovar KR Westbrook GL. The incorporation of NMDA receptors with a distinct subunit composition at nascent hippocampal synapses in vitro. J Neurosci (1999) 19:4180–8. 10234045 10.1523/JNEUROSCI.19-10-04180.1999 PMC6782704

[60] Martel MA Wyllie DJ Hardingham GE. In developing hippocampal neurons, NR2B-containing N-methyl-d-aspartate receptors (NMDARs) can mediate signaling to neuronal survival and synaptic potentiation, as well as neuronal death. Neuroscience (2009) 158:334–43. 10.1016/j.neuroscience.2008.01.080 18378405 PMC2635533

[61] Groc L Heine M Cousins SL Stephenson FA Lounis B Cognet L NMDA receptor surface mobility depends on NR2A-2B subunits. Proc Natl Acad Sci U S A (2006) 103:18769–74. 10.1073/pnas.0605238103 17124177 PMC1693737

[62] Steigerwald F Schulz TW Schenker LT Kennedy MB Seeburg PH Kohr G. C-terminal truncation of NR2A subunits impairs synaptic but not extrasynaptic localization of NMDA receptors. J Neurosci (2000) 20:4573–81. 10844027 10.1523/JNEUROSCI.20-12-04573.2000 PMC6772457

[63] Petralia RS. Distribution of extrasynaptic NMDA receptors on neurons. ScientificWorldJournal (2012) 2012:267120. 10.1100/2012/267120 22654580 PMC3361219

[64] Thomas CG Miller AJ Westbrook GL. Synaptic and extrasynaptic NMDA receptor NR2 subunits in cultured hippocampal neurons. J Neurophysiol (2006) 95:1727–34. 10.1152/jn.00771.2005 16319212

[65] Harris AZ Pettit DL. Extrasynaptic and synaptic NMDA receptors form stable and uniform pools in rat hippocampal slices. J Physiol (2007) 584:509–19. 10.1113/jphysiol.2007.137679 17717018 PMC2277145

[66] Bading H Ginty DD Greenberg ME. Regulation of gene expression in hippocampal neurons by distinct calcium signaling pathways. Science (1993) 260:181–6. 10.1126/science.8097060 8097060

[67] Zhang SJ Steijaert MN Lau D Schutz G Delucinge-Vivier C Descombes P Decoding NMDA receptor signaling: identification of genomic programs specifying neuronal survival and death. Neuron (2007) 53:549–62. 10.1016/j.neuron.2007.01.025 17296556

[68] Zhang SJ Zou M Lu L Lau D Ditzel DA Delucinge-Vivier C Nuclear calcium signaling controls expression of a large gene pool: identification of a gene program for acquired neuroprotection induced by synaptic activity. PLoS Genet (2009) 5:e1000604. 10.1371/journal.pgen.1000604 19680447 PMC2718706

[69] Lau D Bading H. Synaptic activity-mediated suppression of p53 and induction of nuclear calcium-regulated neuroprotective genes promote survival through inhibition of mitochondrial permeability transition. J Neurosci (2009) 29:4420–9. 10.1523/JNEUROSCI.0802-09.2009 19357269 PMC6665744

[70] Leveille F Papadia S Fricker M Bell KF Soriano FX Martel MA Suppression of the intrinsic apoptosis pathway by synaptic activity. J Neurosci (2010) 30:2623–35. 10.1523/JNEUROSCI.5115-09.2010 20164347 PMC2834927

[71] Papadia S Stevenson P Hardingham NR Bading H Hardingham GE. Nuclear Ca^2+^ and the cAMP response element-binding protein family mediate a late phase of activity-dependent neuroprotection. J Neurosci (2005) 25:4279–87. 10.1523/JNEUROSCI.5019-04.2005 15858054 PMC6725111

[72] Chandler LJ Sutton G Dorairaj NR Norwood D. N-methyl d-aspartate receptor-mediated bidirectional control of extracellular signal-regulated kinase activity in cortical neuronal cultures. J Biol Chem (2001) 276:2627–36. 10.1074/jbc.M003390200 11062237

[73] Kim MJ Dunah AW Wang YT Sheng M. Differential roles of NR2A- and NR2B-containing NMDA receptors in Ras-ERK signaling and AMPA receptor trafficking. Neuron (2005) 46:745–60. 10.1016/j.neuron.2005.04.031 15924861

[74] Leveille F El Gaamouch F Gouix E Lecocq M Lobner D Nicole O Neuronal viability is controlled by a functional relation between synaptic and extrasynaptic NMDA receptors. FASEB J (2008) 22:4258–71. 10.1096/fj.08-107268 18711223

[75] Ivanov A Pellegrino C Rama S Dumalska I Salyha Y Ben-Ari Y Opposing role of synaptic and extrasynaptic NMDA receptors in regulation of the extracellular signal-regulated kinases (ERK) activity in cultured rat hippocampal neurons. J Physiol (2006) 572:789–98. 10.1113/jphysiol.2006.105510 16513670 PMC1779993

[76] Wu GY Deisseroth K Tsien RW. Activity-dependent CREB phosphorylation: convergence of a fast, sensitive calmodulin kinase pathway and a slow, less sensitive mitogen-activated protein kinase pathway. Proc Natl Acad Sci U S A (2001) 98:2808–13. 10.1073/pnas.051634198 11226322 PMC30221

