# Supplementary material for: Zika Virus Promotes Neuronal Cell Death in a Non-Cell Autonomous Manner by Triggering the Release of Neurotoxic Factors
Source: Front Immunol. 2017 Aug 23;8:1016. doi: 10.3389/fimmu.2017.01016 (PMC5572413; doi:10.3389/fimmu.2017.01016)
Supplement: Supplementary file 1 [file Data_Sheet_1.DOCX]

Supplementary Material

Zika Virus Promotes Neuronal Cell Death in a Non-cell Autonomous Manner by Triggering the Release of Neurotoxic Factors

Isabella G. Olmo, Toniana G. Carvalho, Vivian V. Costa, Juliana Alves-Silva, Carolina F. Zaniboni, Tatiane C. Izidoro-Toledo, Juliana F. da Silva, Antonio L. Teixeira, Danielle G. Souza, Joao T. Marques, Mauro M. Teixeira, Luciene B. Vieira and Fabiola M. Ribeiro^#^

^*^ *Correspondence:*

Dr. Fabiola M. Ribeiro

fmribeiro@icb.ufmg.br


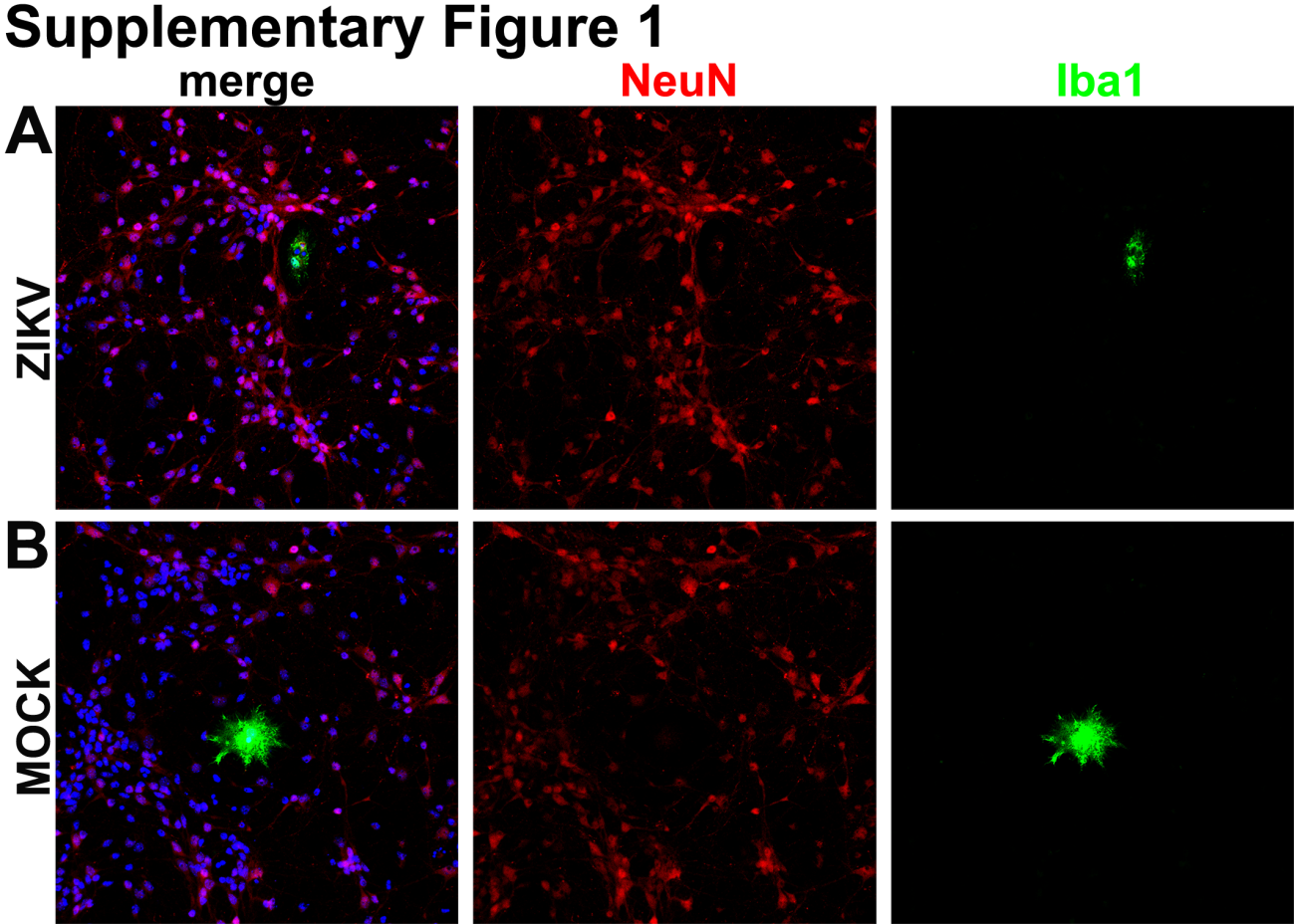


**Supplementary figure 1: Primary corticostriatal cultures are composed predominantly by neurons.** Shown are representative laser-scanning confocal micrographs from primary cultured corticostriatal neurons infected with ZIKV **(A)** or MOCK-infected **(B)** for 48 hours. Immunofluorescence labelling was performed using anti-NeuN (red) and anti-Iba1 (green) antibodies. Cells nuclei are labeled with DAPI (blue). Panels on the left show merged image of all three fluorescent markers. Size bar corresponds to 20 µm in all images.


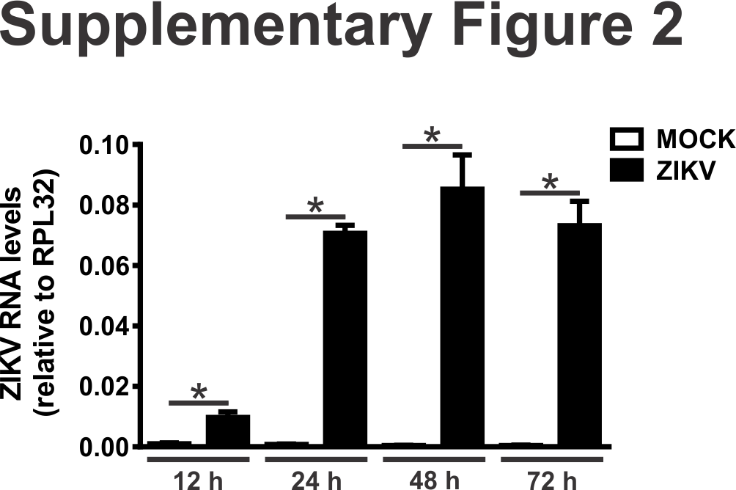


**Supplementary figure 2:** **ZIKV replication levels.** Graph shows ZIKV RNA levels in primary cultured corticostriatal neurons, 12, 24, 48 and 72 hours following MOCK or ZIKV infection. ZIKV RNA levels were assessed by quantitative RT-PCR, which was performed in triplicate and normalized to RPL32 mRNA levels.  Data represent the means ± SEM, n=4. * indicate significant differences (p<0.05).


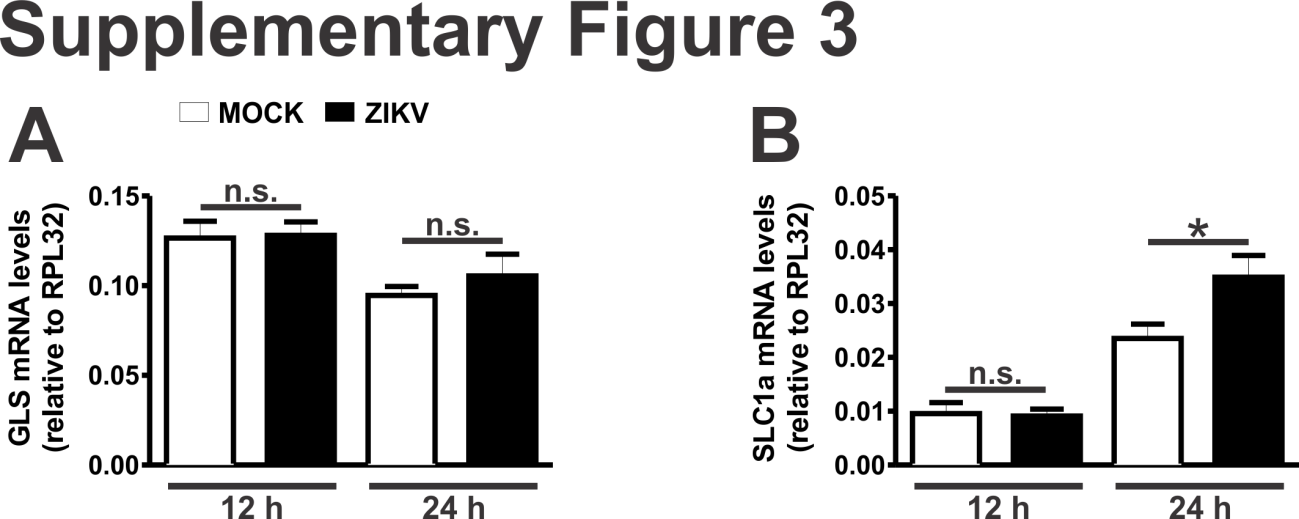


**Supplementary figure 3:** **Effect of ZIKV infection on glutaminase and glutamate transporter expression levels.** Graphs show mRNA levels of glutaminase (GLS) **(A)** and glutamate transporter (SLC1a) **(B)** in primary cultured corticostriatal neurons, 12 and 24 hours following MOCK or ZIKV infection. mRNA levels were assessed by RT-qPCR, which was performed in triplicate and normalized to RPL32 mRNA levels.  Data represent the means ± SEM, n=6. n.s. indicates not significant and * indicates significant difference (p<0.05).
